# Longitudinal antinuclear antibody titers in systemic lupus erythematosus and other rheumatic diseases

**DOI:** 10.3389/fmed.2024.1441221

**Published:** 2024-08-29

**Authors:** Emily A. Littlejohn, Lingxuan Kong, Lu Wang, Emily C. Somers

**Affiliations:** ^1^Cleveland Clinic Rheumatology, Cleveland, OH, United States; ^2^Department of Biostatistics, University of Michigan, Ann Arbor, MI, United States; ^3^Department of Internal Medicine, Environmental Health, and Obstetrics and Gynecology, University of Michigan, Ann Arbor, MI, United States

**Keywords:** ANA testing, longitudinal ANA titers, systemic lupus erythematosus, autoimmune diseases, ANA diseases

## Abstract

**Introduction:**

Antinuclear antibodies (ANAs) are a key feature of systemic lupus erythematosus (SLE) and marker of subclinical autoimmunity. Little is known about longitudinal ANA titers in individuals from the general population or in predicting clinical disease course in persons with rheumatic diseases.

**Methods:**

We performed an exploratory analysis from an academic health system between 1999 and 2020 to assess intra-individual variation in ANAs longitudinally in persons with SLE, other ANA-associated rheumatic diseases, and ANA+ controls without rheumatic disease.

**Results:**

Persons with SLE had a higher odds of positive ANA compared to those with other ANA-associated rheumatic diseases [OR 2.10, 95% CI (1.82, 2.43)] controlling for time and demographics (age, sex, race, ethnicity). Compared to ANA+ controls, the ANA titer strength was significantly higher for both the ANA-associated rheumatic disease (0.33 log units higher) and SLE groups (0.42 log units higher) controlling for demographics and time (*p* < 0.001 for both). Over time from the first positive ANA, titer strength significantly decreased for all three groups, with average monthly decreases ranging between 0.001 to 0.004 log titer units (*p* ≤ 0.001 for all).

**Conclusion:**

Based on this analysis of electronic health data spanning two decades, ANA titers may be more dynamic than previously accepted in patients with SLE and ANA-associated rheumatic diseases, with average titers tending to be higher in early disease and decreasing over time.

## Introduction

Autoantibodies to cellular antigens, commonly referred to as antinuclear (ANAs) or anti-cellular antibodies (1) are a key immunologic feature of systemic lupus erythematosus (SLE). Historically, greater than 95% of SLE patients fulfilling classification criteria for SLE were ANA positive (2). 2019 ACR/EULAR criteria require current or prior ANA positivity as a feature for meeting criteria (3). ANAs are also a marker of subclinical autoimmunity and can be found in healthy individuals with no associated disease. Approximately 13% of the general US adult population (18% of females and 10% of males) are ANA-positive (4) with prevalence increasing with age. Recent studies also show that ANA prevalence has increased over the last three decades in both sexes, although particularly in men, older adults and non-Hispanic whites (5). Risk factors for developing subclinical autoimmunity and predictors for progression to clinical autoimmune phenotypes are poorly understood. An important step is to understand trajectories of ANA positivity and titers within individuals over time, and factors associated with these changes.

ANA testing by indirect immunofluorescence assay (IFA) is a semiquantitative laboratory assay which quantifies the presence of autoantibodies in addition to providing a pattern of nuclear staining. The degree of the ANA titer portends an increased risk for development of autoimmune diseases and research has found that ANA titers are higher in patients with rheumatic diseases than in healthy individuals (6). The presence and activity of autoantibodies have been implicated as a driving mechanism of injury and inflammation in SLE (7, 8). Nevertheless, we have not fully defined the role of these autoantibodies in the general population or in persons with lupus, including the extent to which therapeutics may modulate titers over time.

While a seminal study using the US Department of Defense Serum Repository suggested a progressive accumulation of autoantibodies before the onset of SLE (9), large-scale studies are lacking to assess changes in ANA titers within individuals over time. We performed this study to characterize longitudinal, intra-individual variation in ANAs in persons with SLE or other ANA-associated rheumatic diseases, as well as ANA-positive persons without rheumatic disease, in a large healthcare system in the Midwestern United States.

## Methods

### Study population

Utilizing our academic health center’s electronic health record (EHR) system, with records dating from 1999, we extracted results ANA tests performed by indirect immunofluorescence assay (IFA) for all patients with results from at least two ANA-IFA tests. All testing was performed as part of clinical care by Clinical Laboratory Improvement Amendments (CLIA) certified laboratories. Among these patients, those with at least one positive ANA test were eligible for this study. For this research, we defined an ANA titer of ≥1:80 to as “positive,” and a titer of <1:80 as “negative” (4). This study was approved by the Institutional Review Board of the Cleveland Clinic Foundation.

Among the patients with at least one positive ANA, we screened their electronic medical records for ANA-associated rheumatic disease diagnoses, based on International Classification of Diseases (ICD) coding. Aside from SLE, we considered the following diagnoses to represent “other” ANA-associated rheumatic diseases: scleroderma, Sjogren’s Syndrome, dermatomyositis, polymyositis, mixed connective tissue disease, and undifferentiated connective tissue disease. The ICD-9 and ICD-10 codes utilized to represent the diseases included in this study are listed in Table 1. We then categorized the study population into four groups: (1) SLE in persons with at least one relevant ICD code; (2) A “validated” SLE subset of patients enrolled in the IRB-approved Cleveland Clinic Lupus Cohort (CCLC); (3) “Other” ANA-associated rheumatic disease for patients with at least one ICD code for a non-SLE diagnosis listed above (those who also had an ICD code for SLE were categorized in the SLE group); and (4) ANA-positive controls without a history of the above diseases according to ICD codes. Patients in the validated CCLC (group 2) fulfilled 2012 SLICC and/or 2019 ACR/EULAR classification criteria for SLE (3, 10) as determined by a rheumatologist; this cohort is not a random sample of lupus patients but rather represents a cohort of well-characterized patients with lupus who have provided informed consent for research participation.

**Table 1:** International Classification of Diseases (ICD) codes used for classifying ANA-associated diseases.

Diagnosis	ICD-9	ICD-10
Lupus	710.0	M32.9, M 32.10
Scleroderma	710.1	M34.0, M34.1, M34.9
Sjogren’s syndrome	710.2	M35.0
Dermatomyositis	710.3	M33.9
Polymyositis	710.4	M33.20
Mixed connective tissue disease	710.8	M35.1
Undifferentiated connective tissue disease	710.9	M35.8, 35.9

### Statistical methods

For group comparisons, ANOVA was utilized for continuous variables, and chi-square test for categorical variables. To adjust for confounders, multivariable generalized linear mixed effects models were utilized for analyses with ANA positivity (binary) as the outcome. For analyses with ANA titer strength (magnitude of positivity) as the outcome, multivariable linear mixed effects models were used. As laboratory measurement of ANA titers is based on the detection of “doubling” of levels (eg, 1:80, 1:160, 1:320, etc.), we took a natural logarithm of the ANA titer denominator to normalize the skewness in the distribution of the variable. To account for within-subject correlations across the repeated measurements over time, patient-level random intercepts were placed in the model, and we used these longitudinal random effects models to compare different patient groups and to evaluate the effect of time on the titer value. For multivariable models, the ANA+ control group served as the reference group. Other variables included in the models were: age, sex, race, ethnicity, and time since initial ANA test. In secondary models, we alternatively handled time as time since first rheumatology encounter in our health system. While models utilized all possible ANA measures with exact time point, annual summary graphs utilized the closest ANA measurement to each yearly time point.

## Results

6,983 patients had at least two valid ANA-IFA lab results and thus included in this study (SLE by ICD *n* = 1,665, validated SLE *n* = 71, other ANA-associated rheumatic diagnosis *n* = 1,644, ANA+/no rheumatic diagnosis *n* = 3,603). The median number of ANA assessments per patient was 2 (interquartile range 2, 3) and average follow-up time was 27 months (SD 34). Demographics for the four groups are presented in Table 2. Persons in the validated SLE group tended to be younger, Black race was more common in the SLE groups, and there was a higher proportion of males in the ANA+ control group. 5,546 of the 6,983 patients had a positive ANA at the first observation, where 183 (12.1%) of SLE and 790 (19.4%) of non-SLE had a subsequent negative ANA.

**Table 2:** Demographics according to group.

	SLE – ICD *n* = 1,665	SLE – validated *n* = 71	ANA-associated rheumatic disease *n* = 1,644	ANA+ Control *n* = 3,603	*p*-value ^a^
**Age** (years ± SD)	50.5 ± 15.6	36.5 ± 15.2	50.5 ± 15.6	50.8 ± 18.3	<0.001
**Sex**					
Female Male	1,491 (89.5%) 174 (10.5%)	65 (91.5%) 6 (8.5%)	1,445 (87.9%) 199 (12.1%)	2,868 (79.6%) 735 (20.4%)	<0.001
**Race**					
White Black Other/Unknown	1,092 (65.6%) 446 (26.8%) 127 (7.6%)	44 (62.0%) 26 (36.6%) 1 (1.4%)	1,330 (80.9%) 213 (13.0%) 101 (6.1%)	2,848 (79.0%) 502 (13.9%) 253 (7.0%)	<0.001
**Ethnicity**					
Hispanic Non-Hispanic Unknown	81 (4.9%) 1,472 (88.4%) 112 (6.7%)	1 (1.4%) 69 (97.2%) 1 (1.4%)	35 (2.1%) 1,467 (89.2%) 142 (8.6%)	152 (4.2%) 3,169 (88.0%) 282 (7.8%)	<0.001

Data are presented as mean ± SD or frequency (%) unless otherwise noted.

^a p-value by ANOVA or chi-square test for continuous or categorical variables, respectively.^

**Figure 1:**
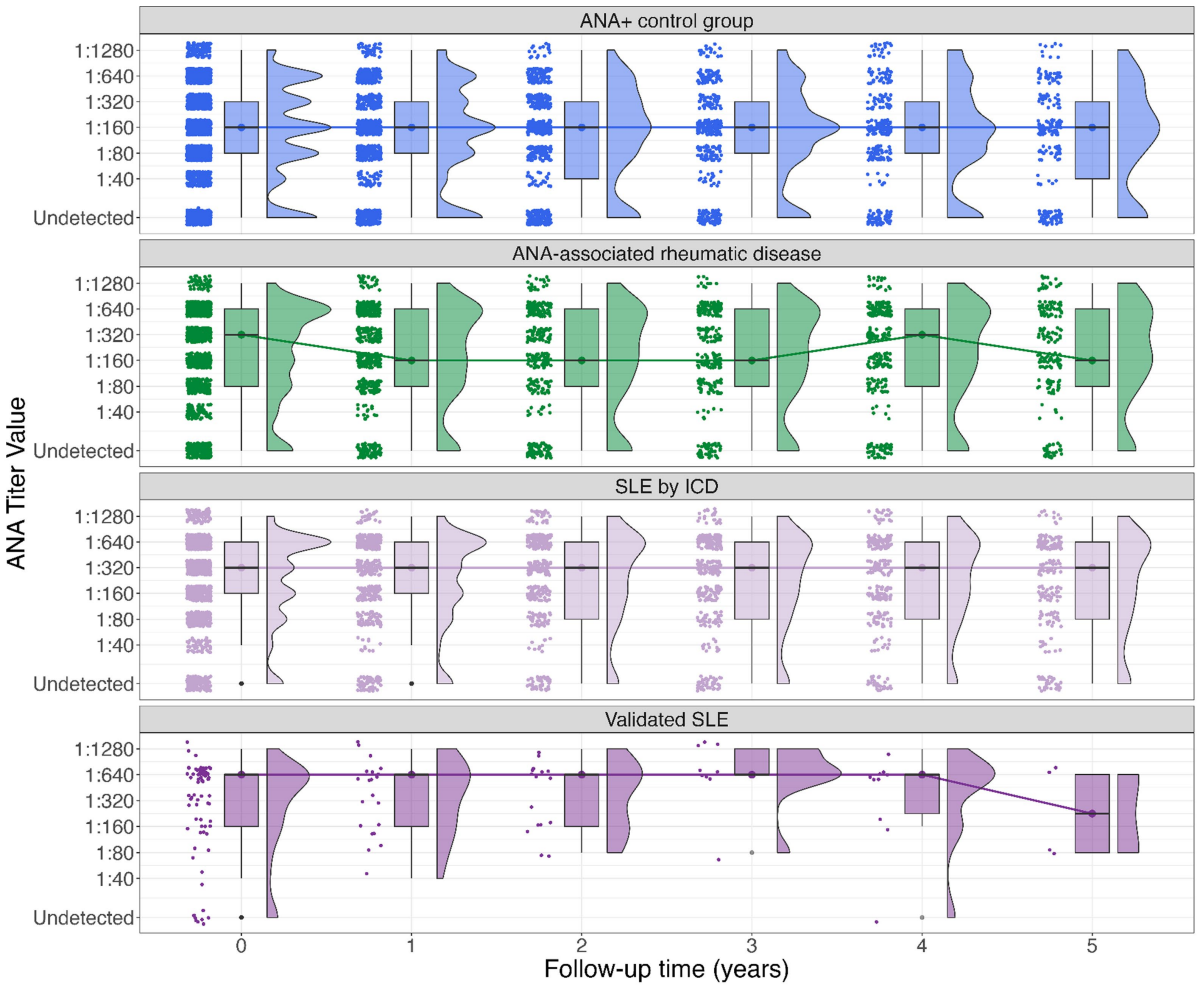
Boxplot of ANA titer among groups (SLE by ICD, validated SLE, other rheumatic diseases, and ANA+ controls). The Y-axis denotes the ANA titer closest to each yearly timepoint*, the X-axis denotes time in years elapsed from baseline (time 0) to 5-year follow-up. Dots and lines denote group medians and their change over time. The upper and lower bound of each box represent the 75 and 25% quantiles, respectively. The histograms to the right side of each box show the frequencies for each titer. *The median elapsed time between each yearly assessment and the actual time of ANA assessment ranged from 1.7 to 4.7 months.

Annual trends for ANA titers are graphically summarized in Figure 1. The median ANA titer over time in the ANA+ control group appears stable at 1:160 over 5 years. In the ANA associated rheumatic disease group the median ANA titer fluctuated between 1:320 and 1:160. ANA values among the persons with SLE were generally higher than for the other rheumatic disease and ANA+ control groups. In the persons with SLE by ICD, the median ANA titer was persistently 1:320, whereas in the validated SLE group the median ANA titer in the first 4 years was 1:640 with a declining trend to 1:160 by year five although sample size is small.

In multivariable modeling, controlling for age, sex, race, and ethnicity (Table 3) SLE patients (combined SLE groups) had a higher odds of a positive ANA on average compared to non-SLE [OR = 2.10, 95% CI (1.82, 2.43)] controlling for time. In SLE (combined groups) the likelihood of having a positive ANA decreased by 0.5% with each month [OR = 0.995, 95% CI (0.992, 0.997)].

**Table 3:** Results from multivariable generalized linear mixed-effects model, with ANA positivity as the binary outcome.

Variable	OR (95% CI)	*P*-value
**Age** (years)	0.998 (0.995, 1.001)	0.28
**Female** (Male referent)	1.32 (1.15, 1.53)	<0.001***
**Race** (White referent)		
Black	0.97 (0.84, 1.12)	0.65
Other/unknown	1.01 (0.80, 1.27)	0.93
**Ethnicity** (non-Hispanic referent)		
Hispanic	0.73 (0.55, 0.98)	0.03*
Unknown	1.55 (1.25–1.91)	<0.001***
**Group** (Non-SLE as referent)		
SLE	2.10 (1.82, 2.43)	<0.001***
**Time from first ANA test (months)**		
Non-SLE	1.002 (1.001, 1.004)	0.003**
SLE	0.995 (0.992, 0.997)	<0.001***

SLE group represents combined SLE groups; SLE based on ICD coding and SLE patients enrolled in the IRB-approved cleveland clinic lupus cohort (CCLC).

Non-SLE referent group represents combined ANA+ controls and ANA-associated disease groups.

In the multivariable linear mixed effects model with log of ANA titer as the outcome (Table 4), at time of first positive ANA test, the average titer strengths (log units) for both the other rheumatic disease and SLE groups were significantly higher compared to the ANA+ controls [coefficients 0.33 (95% CI 0.26, 0.39) and 0.42 (95% CI 0.36, 0.48), respectively; *p* < 0.001 for both], controlling for age, gender, race, and ethnicity. In a separate secondary analysis which was restricted to the SLE and other rheumatic disease groups, similar results were obtained; with other rheumatic disease group as referent, at time of first positive ANA test, the average titer strength for the SLE group was significantly higher [0.08 units higher using the log titer (95% CI 0.002, 0.15, *p* = 0.04)] controlling for age, gender, race, and ethnicity. From the time of the first positive ANA, controlling for all other variables in the model, the ANA titer strength significantly decreased for all three groups, with average monthly decreases ranging between 0.001 to 0.004 in log titer units (*p* < 0.001 for all). In addition, black patients on average had a significantly higher titer compared to white patients [coefficient 0.10 (95% CI 0.03, 0.16); *p* = 0.003]. We performed another secondary analysis using time from the first rheumatology encounter instead of time from first positive ANA test. Results for this model were largely similar to those reported in the primary analysis displayed in Table 4. However, when assessing time from first rheumatology encounter, the only significant association with ANA titer was in the SLE group where there was an average monthly decrease of 0.002 log titer units (95% CI -0.003, −0.001, *p* < 0.001).

**Table 4:** Results from multivariable linear mixed effects models, with log of ANA titer as the outcome.

Variable	Coefficient (95% CI)	*P*-value
**Age** (years)	−0.0003 (−0.002, 0.001)	0.66
**Female** (Male referent)	0.10 (0.03, 0.16)	0.005**
**Race** (White referent)		
Black	0.10 (0.03, 0.16)	0.003**
Other/unknown	−0.06 (−0.16, 0.04)	0.26
**Ethnicity** (non-Hispanic referent)		
Hispanic	0.01 (−0.12, 0.15)	0.85
Unknown	0.20 (0.11, 0.29)	<0.001***
**Group** (ANA+ control referent)		
Other rheumatic disease	0.33 (0.26, 0.39)	<0.001***
SLE	0.42 (0.36, 0.48)	<0.001***
**Time from first positive ANA test (months)**		
ANA+ control	−0.004 (−0.005, −0.004)	<0.001***
Other rheumatic disease	−0.001 (−0.002, −0.001)	<0.001***
SLE	−0.003 (−0.004, −0.003)	<0.001***

SLE group represents combined SLE groups; SLE based on ICD coding and SLE patients enrolled in the IRB-approved Cleveland Clinic Lupus Cohort (CCLC).

## Discussion

The overall prevalence of ANAs in the United States has increased substantially in recent years, rising from 11.0% in 1988–1991 to 15.9% in 2011–2012, which corresponds to 22 and 41 million affected persons, respectively (5). Little is known about what drives the formation of antinuclear antibodies, and in which individuals the accumulation of ANAs is clinically meaningful. Arbuckle et al. (9) demonstrated the accrual of autoantibodies several years in advance of the development of SLE, and thus a subclinical (or preclinical) phase whereby ANAs indicate immune dysregulation and an increased risk for future emergence of disease. Conversely, longitudinal studies of persons with SLE have found that a considerable proportion seroconvert from positive to negative (11–13). Long-term longitudinal data is lacking in ANA-positive individuals, including those with SLE compared to those with other rheumatic diseases or ANA-positive controls without apparent disease. Our data provide initial characterization of these intra-individual trends.

Extending findings of previous studies documenting higher prevalence of ANAs among females (4), we found that among ANA-positive individuals, titer magnitudes tended to be higher among females, when accounting for other demographics and time from first positive ANA. This is consistent with the overall preponderance of autoimmune diseases in women versus men (14). We also found that black patients had a significantly higher titer compared to white patients, which is expected given the increased prevalence of both ANAs (4) and autoimmune diseases such as SLE in the black population (2).

In further analyzing strength of ANA positivity, we found that titers were on average highest for persons with SLE, followed by persons with other ANA-associated rheumatic diseases (Sjogren’s Syndrome, MCTD, polymyositis, dermatomyositis, etc.) and lowest among ANA-positive controls. This suggests a spectrum in ANA levels within all clinically recognized ANA-associated diseases, where the highest titers may be most supportive of a diagnosis of SLE. We found that over time the strength of the ANA titer significantly decreased in the SLE group, as has been corroborated in other studies (11, 12). Frodlund et al. (12) found that 13% of persons with recent onset SLE seroconverted from positive to negative within 8 years, with anti-dsDNA and anti-Smith antibodies as the most frequent nuclear antibodies to decrease over time. Kwon et al. (13) found that in their cohort of 175 persons with SLE with a positive ANA at diagnosis, 9.7% seroconverted to negative over a median of approximately 2 years after diagnosis, with no cases of positive reversion on subsequent ANA tests. Likewise, in the Systemic Lupus International Collaborating Clinics (SLICC) Inception Cohort of patients enrolled within 15 months of SLE diagnosis, ANA seroconversion from positive to negative was reported in 4.8% of the 805 persons with SLE at a follow-up visit occurring between 4–10 years after enrollment (11). There is no clear understanding of what is responsible for these changes or what is reflected by decreasing titers. One longitudinal study found that persons with SLE with at least one negative ANA over the first 5 years of disease had features suggestive of milder disease, with lower activity scores (by SLEDAI-2 K) and fewer autoantibodies to specific antigens compared to patients with persistently positive ANAs (15). This has been corroborated by Kwon et al. (13) who found seroconversion of ANA titer to negative was associated with lower SLE flare risk and increasing ANA titer increment over time was associated with *increased* SLE flare risk. These data suggest ANA titers may reflect a component of underlying disease activity and those with persistently high ANA titers may have a more active SLE phenotype and those with lower ANAs a milder phenotype.

In a study investigating the use of hydroxychloroquine (HCQ) in the preclinical phase of persons who went on to develop SLE, the average number of autoantibodies accrued by the time of diagnosis was lower in patients receiving pre-diagnosis HCQ, than in those who did not receive HCQ (16). This suggests that an increase in autoantibodies, as reflected by the ANA test, may mirror the onset or hastening of symptoms. This study also supports the concept that HCQ can slow or alter the accrual of antibodies. One proposed mechanism is that HCQ alters the pH in intracytoplasmic vesicles and thus the processing and presentation of autoantigenic proteins in MHC class II complexes. This results in a decreased stimulation of CD4+ T cells reactive with self-peptides, decreased release of cytokines, and an overall weakening of the autoimmune process (17).

Understanding what can augment the ANA titer prior to clinical disease would be a next step for investigation. There are several lines of evidence suggesting that ANAs are sensitive to a variety of stimuli including sleep deprivation, parity, exposure to heavy metals, ultraviolet irradiation, and infection (4, 18–23) which may induce change in the underlying autoimmune milieu and potentially influence progression of pre-clinical to clinical SLE.

The strengths of this paper include the large dataset, with longitudinal data spanning over 20 years. ANA-positive patients were stratified by clinical subsets, including those with rheumatic diseases other than SLE and controls without history of a recognized ANA-associated disease. The SLE group in this electronic health record study included a subset of patients with rheumatologist-validation of SLE according to standard classification criteria. To our knowledge, this is one of the first studies that includes longitudinal intra-individual trends of ANA titers versus serial cross-sectional data.

Limitations of our study include the secondary analysis design, utilizing electronic health record data which were not customized to the study question. For instance, there may be a degree of diagnostic misclassification. However, the patients in the prospective Cleveland Clinic Lupus Cohort were all captured in the electronic health records query, demonstrating high sensitivity of the ICD-based query for SLE. Repeat ANA testing was not performed at regularly spaced intervals among individuals; indications for repeat ANA-testing were not routinely captured and physician ordering practices in routine clinical care are not standardized. Reasons for ANA testing are broad and not necessarily based on clinical changes. These unpredictable patterns of testing can lead to various types of biases. Such findings may also have implications for clinical research studies in terms of relevance of ANA positivity as part of eligibility requirements. Authors also note there were demographic differences across groups included in this study; while we adjusted for potential confounders in the multivariable analyses, there may be unmeasured confounders that we were unable to address.

Another limitation that warrants discussion is potential effect of treatment on modulating the ANA and the lack of data on treatment in this study. Our secondary analysis using time from the first rheumatology encounter showed a significant monthly decrease of titer in the SLE group but not other groups. One might infer that disease modifying drugs were started at this first visit, and may play a role in subsequent titer decrease that eventually follows.

Finally, although ANAs are not routinely monitored as an outcome in SLE, therapeutics may impact levels over time (24). However, this EHR-based study is not well-suited to investigating the role of medications; a prospective study with more detailed information on medications and doses (including if prescriptions were dispensed and taken) and ANA assessments at systematic intervals would be needed.

Although the current dogma in rheumatology dictates checking an ANA only once after SLE diagnosis, our data suggest that ANA positivity and magnitude of ANA titer may be more dynamic than broadly recognized. Our findings that ANA titer strength significantly decreased over time from the first positive ANA test in the SLE patient group, aligns with other emerging data. Such declining titers over the course of lupus might also reflect aspects of disease states or flare risk that are beginning to be appreciated given accumulating evidence from recent studies (11, 13, 15). Future research should be aimed at identifying precise interventions which can modulate the accrual of antinuclear antibodies and understanding the interplay between the presence and magnitude of these autoantibodies in association with progression of clinical autoimmune phenotypes.

## Data Availability

The original contributions presented in the study are included in the article/supplementary material, further inquiries can be directed to the corresponding author/s.
